# Comparative effectiveness of antiepileptic drugs in juvenile myoclonic epilepsy

**DOI:** 10.1002/epi4.12349

**Published:** 2019-07-04

**Authors:** Katri Silvennoinen, Nikola de Lange, Sara Zagaglia, Simona Balestrini, Ganna Androsova, Merel Wassenaar, Pauls Auce, Andreja Avbersek, Felicitas Becker, Bianca Berghuis, Ellen Campbell, Antonietta Coppola, Ben Francis, Stefan Wolking, Gianpiero L. Cavalleri, John Craig, Norman Delanty, Michael R. Johnson, Bobby P. C. Koeleman, Wolfram S. Kunz, Holger Lerche, Anthony G. Marson, Terence J. O’Brien, Josemir W. Sander, Graeme J. Sills, Pasquale Striano, Federico Zara, Job van der Palen, Roland Krause, Chantal Depondt, Sanjay M. Sisodiya, the EpiPGX Consortium, Martin J. Brodie, Krishna Chinthapalli, Gerrit‐Jan de Haan, Colin P. Doherty, Sinead Heavin, Mark McCormack, Slavé Petrovski, Narek Sargsyan, Lisa Slattery, Joseph Willis

**Affiliations:** ^1^ Department of Clinical and Experimental Epilepsy UCL Queen Square Institute of Neurology London UK; ^2^ Chalfont Centre for Epilepsy Chalfont St. Peter UK; ^3^ Luxembourg Centre for Systems Biomedicine University of Luxembourg Belvaux Luxembourg; ^4^ Department of Experimental and Clinical Medicine Polytechnic University of Marche Ancona Italy; ^5^ Stichting Epilepsie Instellingen Nederland (SEIN) Heemstede The Netherlands; ^6^ Department of Molecular and Clinical Pharmacology, Institute of Translational Medicine University of Liverpool Liverpool UK; ^7^ The Walton Centre NHS Foundation Trust Liverpool UK; ^8^ Hertie Institute for Clinical Brain Research University of Tübingen Tübingen Germany; ^9^ Belfast Health and Social Care Trust Belfast UK; ^10^ Pediatric Neurology and Muscular Diseases Unit, Department of Neurosciences, Rehabilitation, Ophthalmology, Genetics, Maternal and Child Health University of Genoa Genoa Italy; ^11^ Department of Neuroscience, Reproductive and Odontostomatological Sciences Federico II University Naples Italy; ^12^ Department of Biostatistics University of Liverpool Liverpool UK; ^13^ Molecular and Cellular Therapeutics Royal College of Surgeons in Ireland Dublin Ireland; ^14^ Department of Neurology Beaumont Hospital Dublin Ireland; ^15^ Faculty of Medicine, Division of Brain Sciences Imperial College London UK; ^16^ Department of Genetics University Medical Center Utrecht Utrecht The Netherlands; ^17^ Department of Epileptology University of Bonn Bonn Germany; ^18^ Departments of Neuroscience and Neurology, Central Clinical School Monash University, The Alfred Hospital Melbourne Vic. Australia; ^19^ Pediatric Neurology and Muscular Diseases Unit IRCCS Istituto G. Gaslini Genova Italy; ^20^ Laboratory of Neurogenetics and Neuroscience IRCCS Istituto G. Gaslini Genova Italy; ^21^ University of Twente Enschede The Netherlands; ^22^ Department of Neurology Hôpital Erasme, Université Libre de Bruxelles Brussels Belgium

**Keywords:** seizures, tolerability, adverse drug reactions, valproate

## Abstract

**Objective:**

To study the effectiveness and tolerability of antiepileptic drugs (AEDs) commonly used in juvenile myoclonic epilepsy (JME).

**Methods:**

People with JME were identified from a large database of individuals with epilepsy, which includes detailed retrospective information on AED use. We assessed secular changes in AED use and calculated rates of response (12‐month seizure freedom) and adverse drug reactions (ADRs) for the five most common AEDs. Retention was modeled with a Cox proportional hazards model. We compared valproate use between males and females.

**Results:**

We included 305 people with 688 AED trials of valproate, lamotrigine, levetiracetam, carbamazepine, and topiramate. Valproate and carbamazepine were most often prescribed as the first AED. The response rate to valproate was highest among the five AEDs (42.7%), and significantly higher than response rates for lamotrigine, carbamazepine, and topiramate; the difference to the response rate to levetiracetam (37.1%) was not significant. The rates of ADRs were highest for topiramate (45.5%) and valproate (37.5%). Commonest ADRs included weight change, lethargy, and tremor. In the Cox proportional hazards model, later start year (1.10 [1.08‐1.13], *P* < 0.001) and female sex (1.41 [1.07‐1.85], *P* = 0.02) were associated with shorter trial duration. Valproate was associated with the longest treatment duration; trials with carbamazepine and topiramate were significantly shorter (HR [CI]: 3.29 [2.15‐5.02], *P* < 0.001 and 1.93 [1.31‐2.86], *P* < 0.001). The relative frequency of valproate trials shows a decreasing trend since 2003 while there is an increasing trend for levetiracetam. Fewer females than males received valproate (76.2% vs 92.6%, *P* = 0.001).

**Significance:**

In people with JME, valproate is an effective AED; levetiracetam emerged as an alternative. Valproate is now contraindicated in women of childbearing potential without special precautions. With appropriate selection and safeguards in place, valproate should remain available as a therapy, including as an alternative for women of childbearing potential whose seizures are resistant to other treatments.

**Key Points**

- We conducted a retrospective study of comparative effectiveness of five commonly used antiepileptic drugs in 305 individuals with JME
- Valproate was associated with the highest response rate; levetiracetam ranked second
- Topiramate and valproate were associated with highest rates of ADRs
- Controlling for start year and sex, valproate was associated with the longest treatment duration
- Valproate should remain available as a treatment option for people with refractory JME irrespective of sex

## 1 INTRODUCTION

Juvenile myoclonic epilepsy (JME) is a common epilepsy syndrome, comprising 5%‐10% of all epilepsies.^1^ As JME tends to start during adolescence and lifestyle issues are known to increase the likelihood of seizures, particular attention and care are often required.^2^, ^3^, ^4^ Sodium valproate has long been the antiepileptic drug (AED) of choice for treatment of people with JME, with reported seizure freedom attained in up to 80%.^5^ Despite its effectiveness, valproate use is limited by adverse drug reactions (ADRs), teratogenicity, and neurotoxicity,^6^, ^7^ with important recent restrictions on its use in women of childbearing potential.^8^ Newer AEDs are taking an increasing role in the management of JME, but there are few data on comparative effectiveness to guide treatment choices.^3^, ^9^, ^10^, ^11^, ^12^

People with JME comprised a quarter of those with idiopathic generalized epilepsy (IGE) in the SANAD study, which demonstrated the effectiveness of valproate in IGE overall.^13^ In a retrospective study of 962 individuals with IGE, of whom 357 had JME, valproate monotherapy was associated with a higher response rate compared to lamotrigine or topiramate, but no statistical comparison was undertaken.^9^ Three previous studies have addressed AED comparative effectiveness in JME specifically. These include a prospective study of 156 people^10^ and a retrospective study of 186 individuals^11^; statistical testing of differences between AED effectiveness was not reported in either study.^10^, ^11^ In another retrospective study of 72 individuals, trials with valproate, lamotrigine, or topiramate were associated with better control of myoclonic seizures compared to trials involving phenytoin or carbamazepine.^12^

The prognosis of JME is relatively good with a reported remission rate of approximately 60%,^14^ but a subset of individuals is refractory to appropriate medical treatment.^4^, ^5^, ^14^, ^15^ While AED withdrawal without seizure recurrence may be successful in some,^14^ the majority of people in remission remain on AEDs,^14^, ^15^ raising concerns about long‐term side effects. More information regarding the effectiveness and tolerability of AEDs in JME is required. Our aim was to evaluate AED frequency of use, effectiveness, retention, and tolerability in a real‐world setting. Despite limitations imposed by its observational and retrospective nature, our study provides some much‐needed data on this important topic.

## 2 METHODS

### 2.1 Participants

People with JME were identified from a large clinical database from the EpiPGX consortium, an international multicenter research project on epilepsy pharmacogenetics (www.epipgx.eu). This database contains demographic and clinical details of nearly 10,000 people with a confirmed diagnosis of epilepsy and detailed information on more than 39,000 treatment regimens (hereafter referred to as trials), all collected retrospectively from medical records. Participants were recruited mainly from tertiary referral centers. Data collection was started in 2012 and completed in 2016. Data collection and use was approved by research ethical committees/institutional review boards of each center, and all participants provided written informed consent for appropriately coded use of their clinical data.

The ascertainment of JME cases was based on the criteria of the International League Against Epilepsy (ILAE) and an international consensus statement^2^, ^16^: (a) occurrence of myoclonic seizures, (b) onset between age 8‐25 years, (c) no evidence of progressive disease or intellectual disability, (d) EEG showing generalized epileptiform activity (cases with normal EEG during appropriate AED treatment were included, if deemed to otherwise fulfill criteria for JME by the treating specialist), (e) no clinically significant abnormality on neuroimaging, where available.

**Figure 1:**
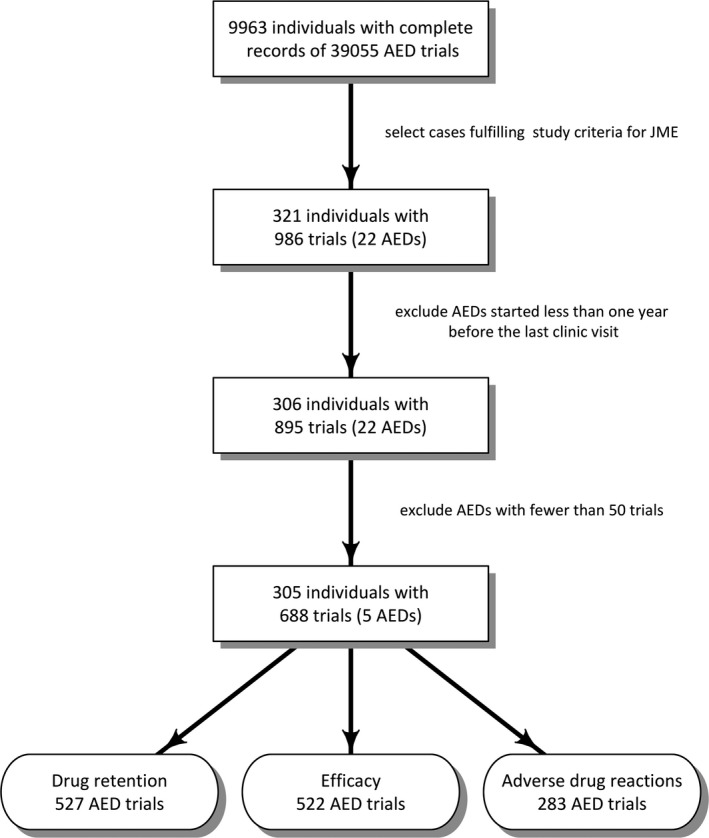
Flowchart for inclusion of people and AED trials. The number of trials for which defined data were available is indicated for each parameter

We identified 321 individuals recruited from specialized epilepsy clinics in Belgium, Germany, Ireland, Italy, the Netherlands, the United Kingdom, and Australia (see Table S1 for details). Among them, 12 (3.7%) had already been included in the SANAD study.^13^ All AED trials from the time of epilepsy diagnosis were considered, including those introduced as add‐on therapy, with some exclusions: (a) AED trials started less than one year before the last clinic visit and (b) AEDs used in fewer than 50 trials. Prescription order, however, was determined considering all AED trials of the individual (see Figure 1). Both regular and extended release AED formulations were included in the analyses.

### 2.2 Outcome measure definition

The classification of AED trial outcomes was modified from the ILAE consensus.^17^ Response was determined clinically as seizure freedom, lasting for ≥12 months, attributable to the AED according to either the treating clinician or the person undertaking phenotyping, or both, and occurring prior to initiation of another treatment for epilepsy. Failure of a trial of treatment was defined as persistent seizures at >50% of the pretreatment seizure frequency despite use of an appropriate AED with an adequate trial. We applied the ILAE criteria for assessing adequacy of an intervention.^17^ To aid outcome assessment, researchers performing the phenotyping used the World Health Organization (WHO) defined daily doses for each AED, as well as the summary table from the ILAE consensus paper defining the minimum dataset to determine whether an intervention is informative.^17^ If data were available but neither criteria for response nor failure were met, the outcome was considered unclassified. If the data required for assessing outcome were missing, the response was categorized as “unknown” (this included trials which were stopped before the outcome was known). Population percentage response was calculated as the number of responses divided by the total number of known outcomes (response, failure, and unclassified). For trials ongoing at last follow‐up, treatment duration was calculated based on the date of last visit, if available. In total, treatment duration was defined for 527 AED trials. Twelve‐month retention rate was defined as the proportion of trials with minimum duration of 12 months. The reason for discontinuation was recorded as due to ADR, lack of effectiveness, ADR and lack of effectiveness, other reason, remission, or unknown. ADRs were classified into nine categories, and their incidence was calculated for each AED as percentage of all trials. Only ADRs considered attributable to the specific AED either by the treating clinician and/or person undertaking phenotyping, or both, were included. The maximum daily dose was recorded for AED trials. Valproate trials were stratified by maximum daily dosage ≤1 g and >1 g, in keeping with classification used in previous literature.^18^

### 2.3 Statistical analyses

Median values were used to express central tendency for durations of trials as data were not normally distributed. Pairwise comparisons with χ^2^ analyses were performed to compare the AEDs with respect to population percentage response rates and rates of ADRs. Similarly, we compared the proportions of females and males receiving valproate, the number of valproate trials between females and males, and the response rates among first, second, or third or later order AED trials. Bonferroni correction for multiple comparisons was applied. We report corrected *P*‐values for simplicity; values ≤0.05 were considered significant.

We modeled discontinuation patterns and compared retention of different AEDs with a Cox proportional hazards model. The outcome measure was trial duration, with hazard ratios presenting hazard of shorter duration. The cofactors included were AED (five levels, one for each AED, with valproate considered the reference level), trial start year (as a continuous variable), and sex (with male sex considered the default). Trial start year and sex were included as we hypothesized that these could influence retention. The global test for nonproportionality was not significant; thus, the Cox model could be applied.

The Mann‐Whitney *U* test was used to compare maximum daily dose distributions of valproate between trials associated with response and trials associated with failure. All analyses were performed using *R*.^19^

## 3 RESULTS

Three hundred and five individuals were included in the final analyses (1). The most commonly used AEDs were valproate, lamotrigine, levetiracetam, carbamazepine, and topiramate, constituting 688 trials (Table 2). Other AEDs were each used in fewer than 50 trials and were excluded from further analyses (see Table S2 and Figure S1 for further demographic and clinical details). Valproate and carbamazepine most often constituted first‐order AED trials, whereas lamotrigine was most commonly started as the second AED trial, levetiracetam as the third trial, and topiramate as the fourth trial (see Tables S3‐S4).

**Table 1:** Demographic and clinical details

Category		
Age (y)	Mean	Range
At last visit	31	13‐78
At epilepsy diagnosis	16	8‐43
At onset	15	8‐25
Epilepsy duration (y)	15	1‐65
AED trials per patient	2	1‐8
	Total number	Percentage (%)
Sex		
Male	95	31.1
Female	210	68.9
Seizure type		
GTCS	267	87.5
Absence	92	30.2
Myoclonic	305	100

Note

Abbreviations: AED, antiepileptic drug; GTCS, generalized tonic‐clonic seizures.

**Table 2:** Details for trials of 22 AEDs in the 306 individuals with treatment trials over 12 mo long

AED name	AED	No. of trials	No. of patients	12‐mo retention rate (%)	Treatment duration (median months ± MAD)	Maximum dose (median mg/d)	AED 1 (%)	AED 2 (%)	AED 3 (%)
Valproate	VPA	279	248	86.1	68 ± 71.5	1200	57.0	20.5	22.5
Lamotrigine	LTG	161	153	83.5	35 ± 35.6	300	26.9	35.2	37.9
Levetiracetam	LEV	124	122	79	31 ± 33.4	2000	10.8	26.4	62.8
Carbamazepine	CBZ	64	62	77.1	30 ± 34.1	800	50.0	22.7	27.3
Topiramate	TPM	60	55	62.7	23 ± 31.1	200	19.1	13.2	67.6
Clobazam	CLB	33	30	47.1	11 ± 13.5	20	2.8	11.1	86.1
Phenobarbital	PB	31	29	66.7	39 ± 106.7	100	37.5	15.6	46.9
Ethosuximide	ESM	27	25	69.2	60 ± 83.7	750	21.4	21.4	57.1
Phenytoin	PHT	27	24	69.2	48 ± 66.7	300	18.5	25.9	55.6
Clonazepam	CNZ	25	22	62.5	21 ± 22.6	4	7.7	26.9	65.4
Zonisamide	ZNS	15	14	86.7	27 ± 22.2	300	–	–	100
Primidone	PRM	12	12	83.3	60 ± 29.3	750	14.3	21.4	64.3
Oxcarbazepine	OXC	11	11	83.3	44 ± 40.4	1350	25.0	41.7	33.3
Acetazolamide	AZM	7	7	0	6 ± 8.6	500	28.6	–	71.4
Gabapentin	GBP	5	5	0	3 ± 3.7	1200	–	40.0	60.0
Diazepam	DZP	3	3	–	–	10	66.7	33.3	–
Vigabatrin	VGB	3	3	50	34 ± 42.9	2000	–	66.7	33.3
Lacosamide	LCM	2	2	100	34 ± 26.4	350	–	–	100
Piracetam	PIR	2	2	0	1 ± 0	2400	–	–	100
Tiagabine	TGB	2	2	–	–	–	–	–	100
Bromide	BRM	1	1	–	–	–	100	–	–
Felbamate	FBM	1	1	100	112 ± 0	1800	–	–	100

Note

The three rightmost columns present the proportions of trials started as the individual's first, second, or third or later AED. Missing data are denoted by (–).

Abbreviations: AED, antiepileptic drug; MAD, median absolute deviation.

### 3.1 Secular patterns of AED trials

**Figure 2:**
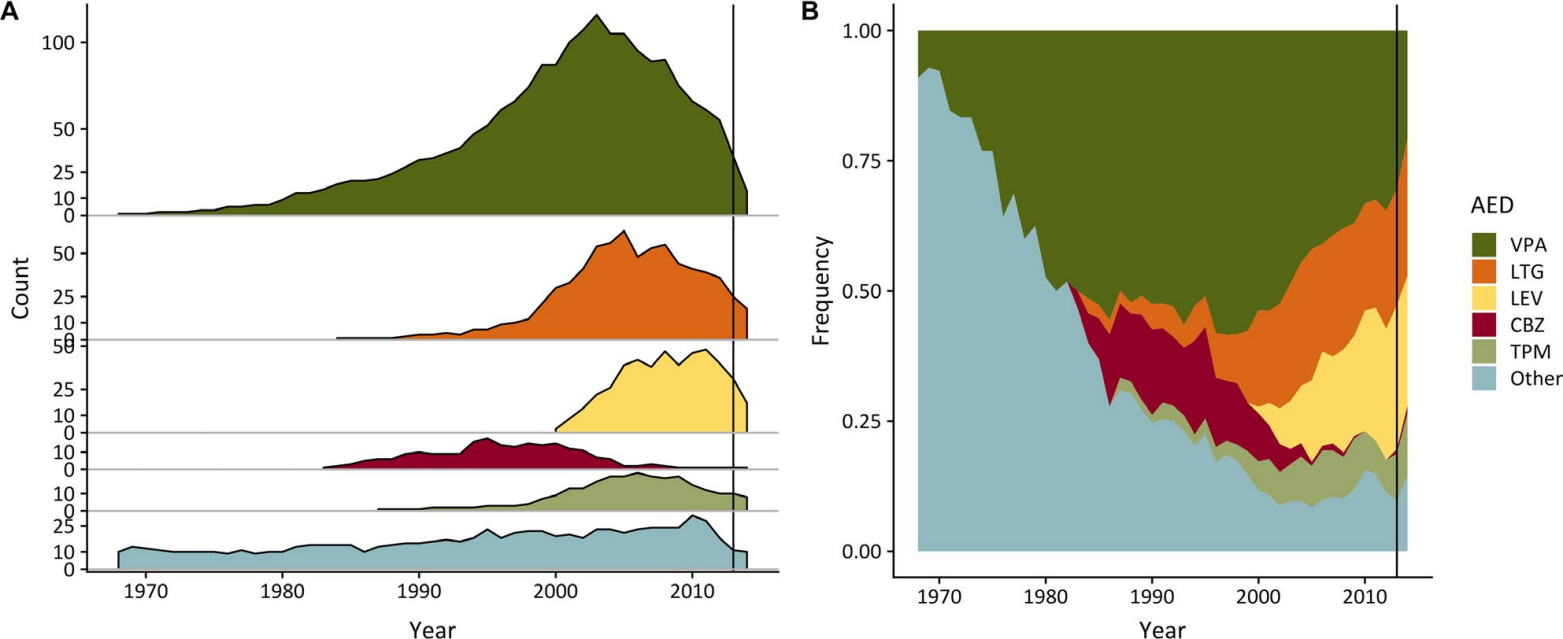
The secular prevalence of AED trials between 1968 and 2014. The extreme right vertical line indicates the 2013 recommendation by the UK Medicines and Healthcare products Regulatory Agency to restrict valproate use, and referral of valproate to the European Medicines Agency Pharmacovigilance Risk Assessment Committee^36^

The first AED trial in the study was started in June 1968 and the last in March 2014. The highest number of trials was recorded in 2003 and 2005, with 206 trials in each. Secular changes in the prevalence of AED trials are presented in Figure 2. Since 2003, there has been a decline in the absolute and relative frequency of valproate trials. During this time, the relative frequency of lamotrigine trials remained stable while the relative frequency of levetiracetam trials increased. The majority of carbamazepine trials took place in the 1990s, with only individual trials recorded from 2005 onwards. See Figure S2 for sex‐specific secular changes in the relative frequencies of AED trials.

### 3.2 Effectiveness

**Figure 3:**
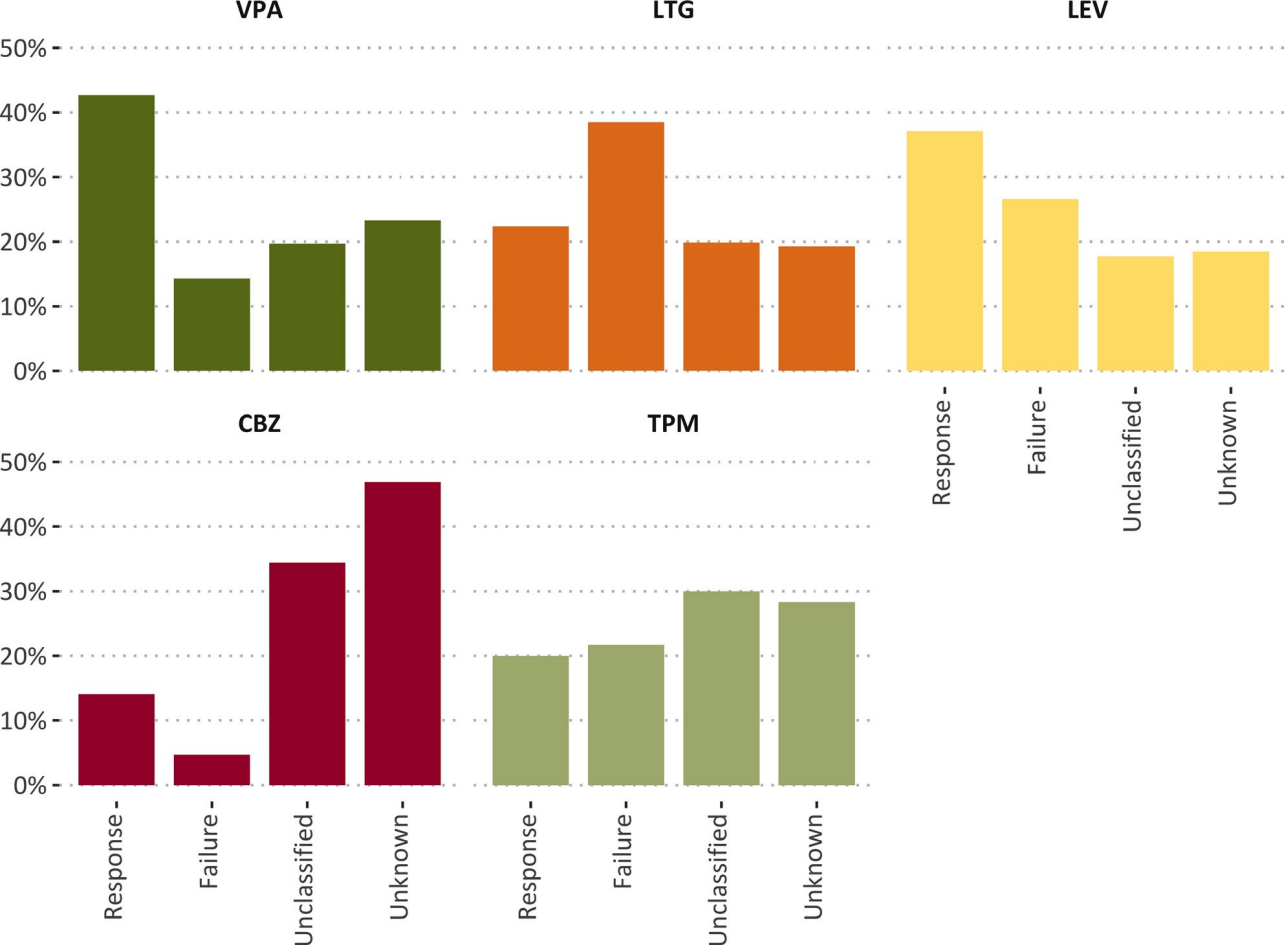
Relative frequencies of trial outcomes for each AED

Response rates to AEDs ranged from 14.1% (carbamazepine) to 42.7% (valproate); see Figure 3 and Table S5 for further details. The population percentage response rate to valproate was significantly higher than the population percentage response rate to lamotrigine (*P* < 0.001), carbamazepine (*P* = 0.03), and topiramate (*P* = 0.02). The differences in population percentage response rates between other AED pairs were not significant (Table S6).

For individual AEDs, correlation between response rate and prescription order could not be performed due to the relatively low response rates observed. Considering all five AEDs together, the response rate of first‐order AED trials was highest (first: 45.6%, second: 38.8% and third or later: 30.0%), but the differences were not statistically significant.

### 3.3 Drug retention

Twelve‐month retention rates ranged from 62.7% for topiramate to 86.1% for valproate; valproate also had the highest median treatment duration (Table 2, Figure S3). In the Cox model, a significant effect of start year on trial duration was observed; later start year was associated with shorter trial duration (HR [CI]: 1.10 [1.08‐1.13], *P* < 0.001). Female sex was associated with shorter trial duration (1.41 [1.07‐1.85], *P* = 0.02). The hazard ratios comparing the duration of the other AEDs to valproate, after adjusting for effect of start year and sex, are presented in Figure S4. Compared to valproate, carbamazepine and topiramate were associated with significantly shorter trial durations.

### 3.4 AED discontinuation

A reason for discontinuation was noted for 69.0% of trials. Lamotrigine had the highest rate of discontinuation due to lack of effectiveness (40.8% of trials), but a low rate of discontinuation due to ADRs (10.2%). The respective figures for levetiracetam were 25.4% and 14.9%. Topiramate was associated with the highest rate of discontinuation due to ADRs (24.5%) (Table S7).

### 3.5 Adverse drug reactions

The frequency of ADRs ranged from 14.5% for carbamazepine to 45.5% for topiramate. The rate of ADRs for carbamazepine was significantly lower than the rate of ADRs for topiramate (*P* = 0.005) and valproate (*P* = 0.010). The rate of ADRs for lamotrigine was also significantly lower than that for topiramate (*P* < 0.001) and valproate (*P* < 0.001). Overall, the three most common ADRs were weight change (reported in 64 trials), lethargy (40 trials), and tremor (37 trials). The incidence of specific ADRs, however, varied for each AED (see Table 3). Additional information on ADRs is presented in Appendix S1.

**Table 3:** Incidence of the nine most frequent adverse drug reactions (ADRs) for each antiepileptic drug (AED), expressed as absolute number of trials with ADR, and as percentage of all trials

AED	Patients with ADR (%)	Weight change	Lethargy	Tremor	Cognitive impairment	Behavioral disorder	Depression	Gastrointestinal ADRs	Adverse cutaneous reaction	Speech disorder
VPA	93 (37.5)	58 (20.8)	19 (6.8)	30 (10.8)	12 (4.3)	6 (2.2)	2 (0.7)	3 (1.1)	0 (0.0)	1 (0.4)
LTG	25 (16.3)	2 (1.2)	4 (2.5)	4 (2.5)	3 (1.9)	0 (0.0)	2 (1.2)	2 (1.2)	4 (2.5)	0 (0.0)
LEV	30 (24.6)	0 (0.0)	11 (8.9)	1 (0.8)	3 (2.4)	16 (12.9)	4 (3.2)	0 (0.0)	0 (0.0)	1 (0.8)
CBZ	9 (14.5)	0 (0.0)	1 (1.6)	0 (0.0)	0 (0.0)	0 (0.0)	0 (0.0)	4 (6.2)	4 (6.2)	0 (0.0)
TPM	25 (45.5)	4 (6.7)	5 (8.3)	2 (3.3)	10 (16.7)	3 (5.0)	3 (5.0)	0 (0.0)	0 (0.0)	3 (5.0)

### 3.6 Valproate dosage and use in males and females

Of the 119 valproate trials associated with response, 61 (51.3%) involved a maximum daily dose of ≤1 g. In trials associated with response, the median maximum daily dose was 1000 mg/d, whereas for failed trials, it was 1500 mg/d. The difference in the maximum daily dose distributions was statistically significant (Mann‐Whitney *P* = 0.004; see Figure S5).

Valproate was trialed in 92.6% of males and 76.2% of females; the difference was significant (*P* = 0.001). Females more frequently had interruption of valproate treatment, with more than one valproate trial in 13.8% of females versus 10.2% of males.

## 4 DISCUSSION

We explored the use of AEDs in 305 people with JME over a long period, providing observational real‐world insight into the tolerability and effectiveness of AEDs in this syndrome. The topic is timely as means of managing risks of valproate have recently undergone review by the European Medicines Agency,^8^ with important implications for management of JME. Prospective JME‐specific trials involving the current array of AEDs, including valproate, are now very unlikely, and observational studies such as ours provide important information for clinical practice. Compared to previous JME‐specific studies,^10^, ^11^, ^12^ a strength of our study is a relatively large number of trials with AEDs other than valproate, formal comparative testing, and stringent criteria for JME diagnostic ascertainment. We applied a uniform method of data collection in all centers; data were stored in a single database. Recently, we successfully applied the same strategy for investigating AED use in mesial temporal lobe epilepsy.^20^

An obvious weakness of our study is its retrospective design: We could not account for possible effects of the natural history of JME on outcomes. Patients were gathered mainly from tertiary centers, which should increase confidence in the validity of syndromic diagnosis.^21^ Individuals with refractory epilepsy may, however, be overrepresented in our sample. Other JME studies report a female preponderance.^15^ In our study, the more pronounced gender difference may further reflect referral bias to our tertiary centers, related, for example, to management issues around pregnancy or family planning. Observed patterns of AED use may be biased by local practices at the participating centers. EpiPGX was not designed to look prospectively at neurodevelopmental outcomes in children exposed to AEDs in utero, and we cannot comment on this very important topic. Lastly, classifying drug response is challenging, with various schemes: We chose a modification of the ILAE scheme.^17^ The ILAE definition of seizure freedom requires that duration of seizure freedom is three times the previous interseizure interval (“Rule of Three”) or at least 12 months, whichever is longer.^17^ The limitations of the “Rule of Three” for prediction of ongoing seizure freedom are, however, recognized.^22^ The conventional definition based only on at least 12 months' seizure freedom is still commonly used.^23^ The ILAE definition of treatment failure is lack of seizure freedom after an informative trial of an intervention^17^; for clinical utility, we defined failure as less than 50% reduction in seizure frequency.

The frequencies of trials for individual AEDs are influenced by changes in AED availability over time. The predominance of valproate was expected considering its long‐standing availability and typical practice having been to use it as first‐line treatment for JME.^24^ Carbamazepine, which is not recommended for treatment of JME,^3^ emerged as the fourth most commonly tried AED. In over half of these trials, carbamazepine was the first AED tried. Most carbamazepine trials in our sample date to the 1990s, when fewer treatment options were available. In some individuals, carbamazepine trials may also have predated their JME diagnosis.

The response rate to valproate was the highest, and the population percentage response rate differed significantly from that of lamotrigine, carbamazepine, and topiramate. Levetiracetam had the second highest response rate with no significant difference compared to valproate. Effectiveness measures may be affected by prescription order^25^; most trials with levetiracetam were the individual's third or later order AED trial. The comparison of response rates is limited by lack of data on whether an AED was introduced as monotherapy or as add‐on. This would have been particularly interesting for valproate and lamotrigine, given the evidence for synergism between these AEDs.^26^ Further limitations are the high frequencies of unclassified and unknown outcomes. We lacked effectiveness information for specific seizure types; we expect that this may impact upon rating for absence and myoclonic seizures especially. According to previous reports, carbamazepine and lamotrigine are associated with risk of more frequent myoclonic seizures,^3^, ^27^ but we were unable to assess whether this could have affected our outcomes. Nevertheless, our results are in keeping with prospective data on the high effectiveness of valproate in the management of IGE.^13^ They also indicate levetiracetam as an effective alternative, in keeping with previous reports of effectiveness of levetiracetam as add‐on treatment^28^ or monotherapy^29^ in JME.

Pseudoresistance, that is treatment failure caused by lifestyle factors such as alcohol consumption and sleep deprivation, is a recognized concept in JME.^4^ As a further limitation, we were unable to assess whether such factors contributed to our treatment outcomes.

Valproate was associated with the highest 12‐month retention rate and median treatment duration. In our survival analysis, valproate was associated with a significantly longer trial duration compared to carbamazepine or topiramate, but not lamotrigine or levetiracetam. Retention parameters may be skewed by older trials started when fewer alternative AEDs were available. This was confirmed in the survival analysis. Another possible source of bias is the effect of prescription order, as an individual's first AED trial is more likely to be successful,^25^ and therefore have longer duration, compared to subsequent trials. In our sample, valproate most commonly constituted an individual's first trial.

The highest rate of ADRs was observed for topiramate, with valproate ranking second. For both AEDs, rates of ADRs were similar to those observed in SANAD.^13^ While there is some evidence that topiramate may be better tolerated than valproate as monotherapy for JME,^30^ our contrasting findings may reflect effects of polytherapy.^31^ Due to the retrospective nature of our study, some types of ADRs may have been more frequently recorded than others. The commonest ADR to topiramate was cognitive impairment, whereas for valproate, weight change was most common. These findings are generally in keeping with prospectively collected data.^13^, ^32^ The commonest ADR to levetiracetam was behavioral disorder, and indeed concerns have been previously raised about the neuropsychiatric side effects of levetiracetam and topiramate in people with JME,^33^ in whom psychiatric comorbidities and impulsive personality traits appear overrepresented.^34^

No valproate dose is considered safe in pregnancy, but some risks associated with fetal exposure to valproate are known to be dose‐dependent,^6^ as are some of the valproate‐associated ADRs.^7^ Recommended maintenance doses for valproate are 1‐2 g/d.^35^ There are suggestions that daily monotherapy doses of ≤1 g are sufficient for maintaining seizure freedom for a significant proportion of people with JME.^10^, ^18^ In our group, over half of successful valproate trials involved maximum doses of 1 g/d. Together with previous reports, our findings suggest that in people with JME for whom valproate is a necessary and appropriate treatment choice, it is reasonable to aim initially for lower doses.

Valproate is being superseded by other AEDs (see Figure 2). The onset of this change in 2003 precedes the more recent regulatory and pharmacovigilance measures,^8^, ^36^ and likely reflects the combination of accumulating evidence of adverse outcomes in valproate‐associated pregnancies^6^ and increasing availability of alternative AEDs. We also observed sex differences in the patterns of valproate use: Males were significantly more likely to receive valproate than females, and females' valproate trials were more likely to be subject to interruptions. These findings are in keeping with population‐based reports of decline in valproate use in treatment of epilepsy in women.^37^ While these observations reflect serious concern over adverse effects, teratogenicity, and risk of neurodevelopmental disorders in exposed offspring,^6^ they also suggest that people with JME, especially female, may be deprived of the most effective treatment for their condition, a concern already voiced by others.^11^ Poor seizure control carries well‐recognized risks,^38^ and it is important to consider the possible effects of reduced valproate use on seizure control and other outcomes in JME. Current restrictions on valproate use^8^ warrant a re‐assessment of therapeutic options for JME. Valproate was associated with a considerable rate of ADRs, and its potential for teratogenicity and inducing neurodevelopmental disorders has significant implications for patient choice, counseling and treatment monitoring. Data on pregnancy‐related risks associated with valproate use have been widely disseminated again recently,^8^ and new measures for their management are in place. In girls and women, valproate use must take these new measures into account.^8^ Among other AEDs, our results corroborate the role of levetiracetam in the management of JME. Based on its high retention and response rates, however, valproate should remain available, with the necessary counseling and safeguards, as an alternative for people not responsive to other treatments, irrespective of sex.

## CONFLICTS OF INTEREST

AA is employed by UCB Pharma, Belgium as Associate Director. AC reports grants from Eisai outside the submitted work. JC reports personal fees from UCB Pharma, personal fees from Sanofi‐Synthelabo, personal fees from Glaxo Smith Kline, personal fees from Janssen‐Cilag, personal fees from Pfizer and personal fees from Eisai to undertake lectures, participate in advisory boards and to undertake research. HL has received honoraria for consulting or speaking or travel support from Bial, BioMarine, Desitin, Eisai, and UCB, and research support from Bial, all outside the submitted work. JWS has received research funding from Eisai, and UCB, personal fees from Eisai, Bial, Janssen and UCB outside the submitted work. In the past 36 months, GJS has received personal fees for consulting and/or speaking from UCB Pharma and Eisai, all outside the submitted work. CD has received honoraria and grant funding from UCB, unrelated to the current study. SMS reports representing the Association of British Neurologists and The Royal College of Physicians (London) at the MHRA Valproate Stakeholders Network, is a member of the scientific advisory board of Dravet Syndrome UK, patron of AHC UK. SMS has received honoraria or grant funding from UCB, Eisai, Vitaflo and Nutricia, all outside the submitted work. The remaining authors have no conflicts of interests. We confirm that we have read the Journal's position on issues involved in ethical publication and affirm that this report is consistent with those guidelines.

## Data Availability

Requests for data may be addressed to the EpiPGX steering committee via the corresponding author.

## APPENDIX 1

### EpiPGX Contributors

Martin J. Brodie, Krishna Chinthapalli, Gerrit‐Jan de Haan, Colin P. Doherty, Sinead Heavin, Mark McCormack, Slavé Petrovski, Narek Sargsyan, Lisa Slattery, Joseph Willis
