# Supplementary material for: Comparative effectiveness of antiepileptic drugs in juvenile myoclonic epilepsy
Source: Epilepsia Open. 2019 Jul 4;4(3):420–30. doi: 10.1002/epi4.12349 (PMC6698679; doi:10.1002/epi4.12349)
Supplement: Supplementary file 1 [file EPI4-4-420-s001.docx]

Supplementary material for “Comparative effectiveness of antiepileptic drugs in juvenile myoclonic epilepsy”

Katri Silvennoinen, Nikola de Lange, Sara Zagaglia, Simona Balestrini, Ganna Androsova, Merel Wassenaar, Pauls Auce, Andreja Avbersek, Felicitas Becker, Bianca Berghuis, Ellen Campbell, Antonietta Coppola, Ben Francis, Stefan Wolking, Gianpiero L Cavalleri, John Craig, Norman Delanty, Michael R Johnson, Bobby PC Koeleman, Wolfram S Kunz, Holger Lerche, Anthony G Marson, Terence J O’Brien, Josemir W Sander, Graeme J Sills, Pasquale Striano, Federico Zara, Job van der Palen, Roland Krause^, Chantal Depondt^, Sanjay M Sisodiya^, the EpiPGX Consortium

## Supplementary methods

### AED prescription order

The order of prescription was recorded for AED trials, where possible. The start date was known for 584 (84.9%) trials. For participants with undocumented start dates for some AED trials, trials with missing start date were sorted by rank of entry and were considered to have occurred before trials with known start dates.
For each AED, data on individual participants’ order of prescription were used to calculate relative frequencies of use as 1) first to third or later AED and 2) first to tenth or later AED. Results for the former were also stratified by sex.

## Supplementary results

### Contributing centers and patient characteristics

The initial 321 individuals identified from the EpiPGX cohort were compiled from 9 centers (Supplementary Table 1).

Supplementary Table 1: Participants by center. Total count and percentage among all included individuals are displayed for each center.

|  | Total Count | Percentage |
| --- | --- | --- |
| Eberhard-Karl-Universität, Tübingen, Germany | 125 | 41.0 |
| Universitair Medisch Centrum Utrecht, Netherlands | 66 | 21.6 |
| The University of Liverpool, United Kingdom | 33 | 10.8 |
| Université Libre de Bruxelles, Belgium | 25 | 8.2 |
| University College London, United Kingdom | 21 | 6.9 |
| Istituto Giannina Gaslini, Genoa, Italy | 20 | 6.6 |
| Royal College of Surgeons in Ireland | 19 | 6.2 |
| Stichting Epilepsie Instellingen Nederland, Netherlands | 9 | 3.0 |
| The University of Melbourne, Australia | 3 | 1.0 |

The degree of female preponderance varied between centers (Supplementary Table 2).

Supplementary Table 2: Sex distribution of individuals by syndromic diagnosis and recruiting center. Sex distribution at each center is specified for 1) all people with epilepsy included in the study, 2) individuals with IGE, and 3) individuals with JME. Figures are given as total count and percentage among syndromic group.

|  | Male total | Female total | Male IGE | Female IGE | Male JME | Female JME |
| --- | --- | --- | --- | --- | --- | --- |
| Eberhard-Karl-Universität, Tübingen, Germany | 520 (40.7%) | 758 (59.3%) | 65 (32.3%) | 136 (67.7%) | 40 (32.0%) | 85 (68.0%) |
| Universitair Medisch Centrum Utrecht, Netherlands | 131 (36.4%) | 229 (63.6%) | 20 (25.6%) | 58 (74.4%) | 18 (27.3%) | 48 (72.7%) |
| The University of Liverpool, United Kingdom | 1232 (52.1%) | 1133 (47.9%) | 35 (42.2%) | 48 (57.8%) | 15 (45.5%) | 18 (54.5%) |
| Université Libre de Bruxelles, Belgium | 414 (47.0%) | 467 (53.0%) | 16 (44.4%) | 20 (55.6%) | 7 (28.0%) | 18 (72.0%) |
| University College London, United Kingdom | 1390 (46.4%) | 1604 (53.6%) | 48 (40.3%) | 71 (59.7%) | 4 (19.0%) | 17 (81.0%) |
| Istituto Giannina Gaslini, Genoa, Italy | 262 (45.0%) | 320 (55.0%) | 29 (34.5%) | 55 (65.5%) | 10 (40.0%) | 15 (60.0%) |
| Royal College of Surgeons in Ireland | 579 (49.2%) | 597 (50.8%) | 12 (33.3%) | 24 (66.7%) | 6 (31.6%) | 13 (68.4%) |
| Stichting Epilepsie Instellingen Nederland, Netherlands | 1271 (52.7%) | 1141 (47.3%) | 11 (61.1%) | 7 (38.9%) | 4 (44.4%) | 5 (55.6%) |
| The University of Melbourne, Australia | 315 (55.9%) | 249 (44.1%) | 4 (50.0%) | 4 (50.0%) | 1 (33.3%) | 2 (66.7%) |
| Belfast Health and Social Care Trust, United Kingdom | 0 (0%) | 168 (100%) | 0 (0%) | 11 (100%) | 0 (0%) | 0 (0%) |
| Universitätsklinikum Bonn, Germany | 17 (50.0%) | 17 (50.0%) | 0 (0%) | 2 (100%) | 0 (0%) | 0 (0%) |

At last visit, the eldest individuals were in their eighth decade (Supplementary Figure 1).


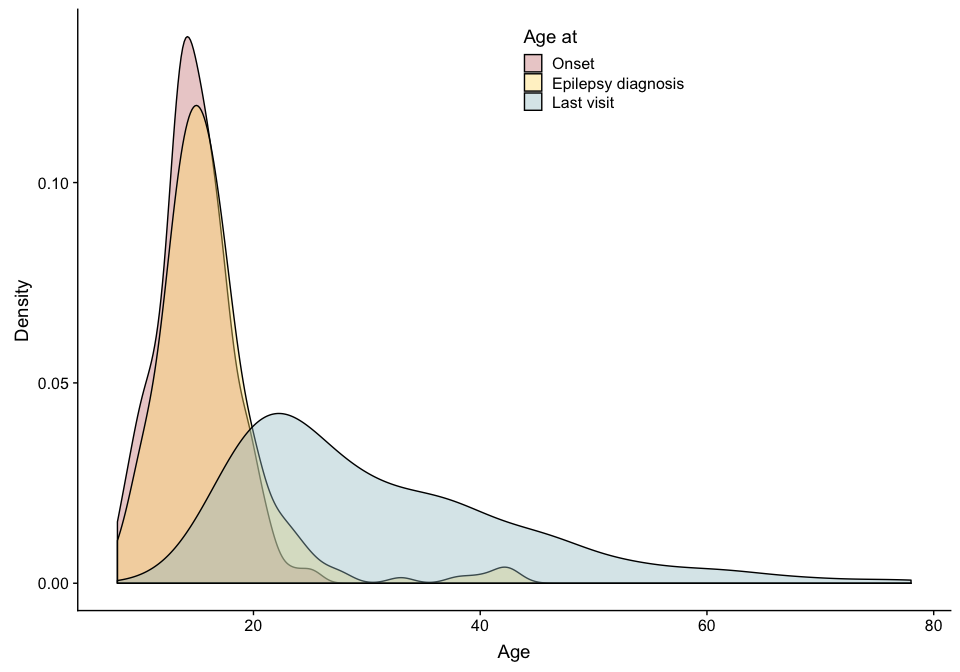


Supplementary Figure 1: Distribution of participants’ ages at onset, diagnosis and last visit.

###

### Order of AED trials

Supplementary Table 3: AED prescription order. Columns N1-N10 correspond to AED trial order for individual participants in the study. Their relative frequency (in percentage) is presented for each AED.

| AED | N 1 | N 2 | N 3 | N 4 | N 5 | N 6 | N 7 | N 8 | N 9 | N 10 |
| --- | --- | --- | --- | --- | --- | --- | --- | --- | --- | --- |
| VPA | 57.0 | 20.5 | 9.1 | 5.4 | 3.7 | 1.7 | 1.0 | 0.3 | 0.3 | 1.0 |
| LTG | 26.9 | 35.2 | 18.1 | 8.2 | 4.4 | 3.3 | 0.5 | 2.2 | 0.5 | 0.5 |
| LEV | 10.8 | 26.4 | 29.1 | 12.8 | 6.8 | 4.1 | 2.0 | 3.4 | 2.0 | 2.7 |
| CBZ | 50.0 | 22.7 | 13.6 | 7.6 | 4.5 | 1.5 | - | - | - | - |
| TPM | 19.1 | 13.2 | 14.7 | 14.7 | 10.3 | 8.8 | 11.8 | 4.4 | - | 2.9 |

Supplementary Table 4: AED order of treatment by sex. The percentage represents the share of an AED given as first, second, or third or later prescription out of the total prescriptions for this AED, and is presented separately for both sexes.

| AED | AED1 Male (%) | AED2 Male (%) | AED3+ Male (%) | AED1 Female (%) | AED2 Female (%) | AED3+ Female (%) |
| --- | --- | --- | --- | --- | --- | --- |
| VPA | 60.7 | 21.5 | 17.8 | 55.0 | 19.9 | 25.1 |
| LTG | 13.9 | 27.8 | 58.3 | 30.1 | 37.0 | 32.9 |
| LEV | 4.9 | 31.7 | 63.4 | 13.1 | 24.3 | 62.6 |
| CBZ | 63.6 | 4.5 | 31.8 | 43.2 | 31.8 | 25.0 |
| TPM | 20.8 | 12.5 | 66.7 | 18.2 | 13.6 | 68.2 |

### AED usage over time by sex

The past two decades show a decrease in the relative frequency of females’ valproate trials, whereas for males, the relative frequency of valproate trials showed a stable or slightly increasing trend. At the same time, the relative frequency of lamotrigine trials showed an increase in females, while this was not seen in males (Supplementary Figure 2).


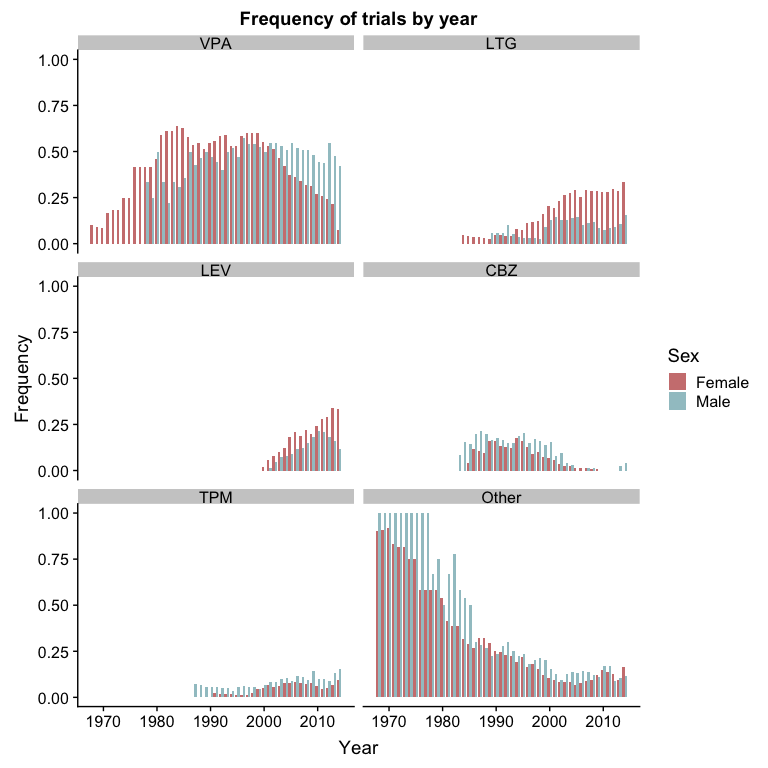


Supplementary Figure 2: Relative frequencies of AED trials by sex and year (range 1968-2014).

### Trial outcome

Among the AEDs, the response rate to levetiracetam was second highest, while lamotrigine ranked third (Supplementary Table 5). Lamotrigine had the highest failure rate of 38.5%; the failure rate of levetiracetam was second highest. Carbamazepine had the highest rate of unknown and unclassifiable outcomes, whereas both response and failure rates were lowest among the AEDs. The second highest rates of unknown and unclassifiable outcomes were observed for topiramate trials.

Supplementary Table 5: AED response summary. For each AED, the total number of trials associated with each outcome is given. This is also expressed as percentage of all trials.

| AED | Response | Failure | Unclassified | Unknown | Total |
| --- | --- | --- | --- | --- | --- |
| VPA | 119 (42.7%) | 40 (14.3%) | 55 (19.7%) | 65 (23.3%) | 279 |
| LTG | 36 (22.4%) | 62 (38.5%) | 32 (19.9%) | 31 (19.3%) | 161 |
| LEV | 46 (37.1%) | 33 (26.6%) | 22 (17.7%) | 23 (18.5%) | 124 |
| CBZ | 9 (14.1%) | 3 (4.7%) | 22 (34.4%) | 30 (46.9%) | 64 |
| TPM | 12 (20.0%) | 13 (21.7%) | 18 (30.0%) | 17 (28.3%) | 60 |

The population percentage response rates for each AED and their pairwise comparison are shown in Supplementary Table 6. Valproate had the highest population percentage response rate of 55.6%; this was significantly higher compared to lamotrigine, carbamazepine, and topiramate, but not levetiracetam.

Supplementary Table 6: Pairwise p values for comparison of population percentage response rates among the AEDs.

| AED | VPA | LEV | TPM | LTG | CBZ | Rate |
| --- | --- | --- | --- | --- | --- | --- |
| VPA | 1.0000 | 1.0000 | 0.0164 | 0.0000 | 0.0295 | 55.6% |
| LEV | 1.0000 | 1.0000 | 0.7357 | 0.0749 | 0.7907 | 45.5% |
| TPM | 0.0164 | 0.7357 | 1.0000 | 1.0000 | 1.0000 | 27.9% |
| LTG | 0.0000 | 0.0749 | 1.0000 | 1.0000 | 1.0000 | 27.7% |
| CBZ | 0.0295 | 0.7907 | 1.0000 | 1.0000 | 1.0000 | 26.5% |

### Duration of treatment

Treatment durations showed non-normal distribution, with a wide range especially for females’ valproate trials (Supplementary Figure 3).


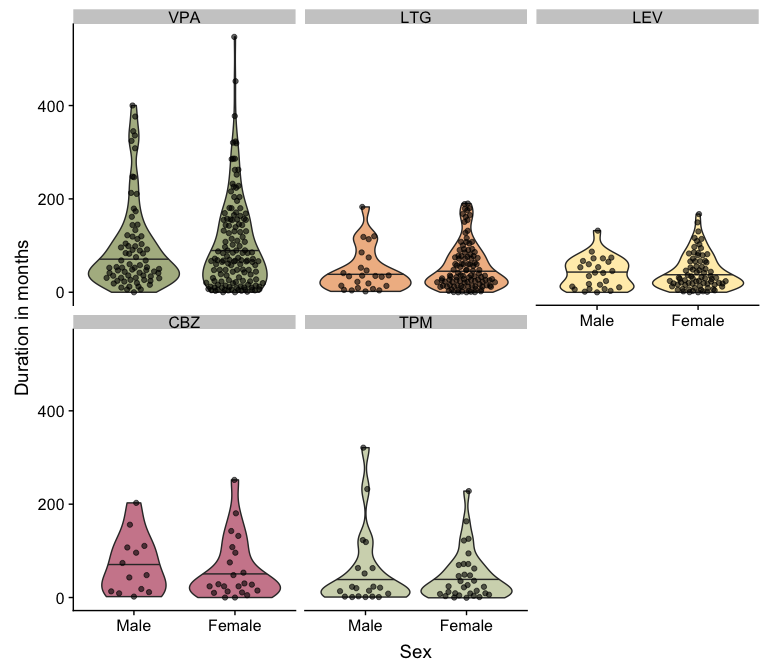


Supplementary Figure 3: AED treatment duration for each AED, stratified by sex. The horizontal bar presents median duration while the violin plot displays the density of trials according to duration. Individual trials are represented by points.

###

### AED retention

Compared to valproate, trials of carbamazepine and topiramate had a significantly shorter retention time when adjusted for start year and sex (Supplementary Figure 4).


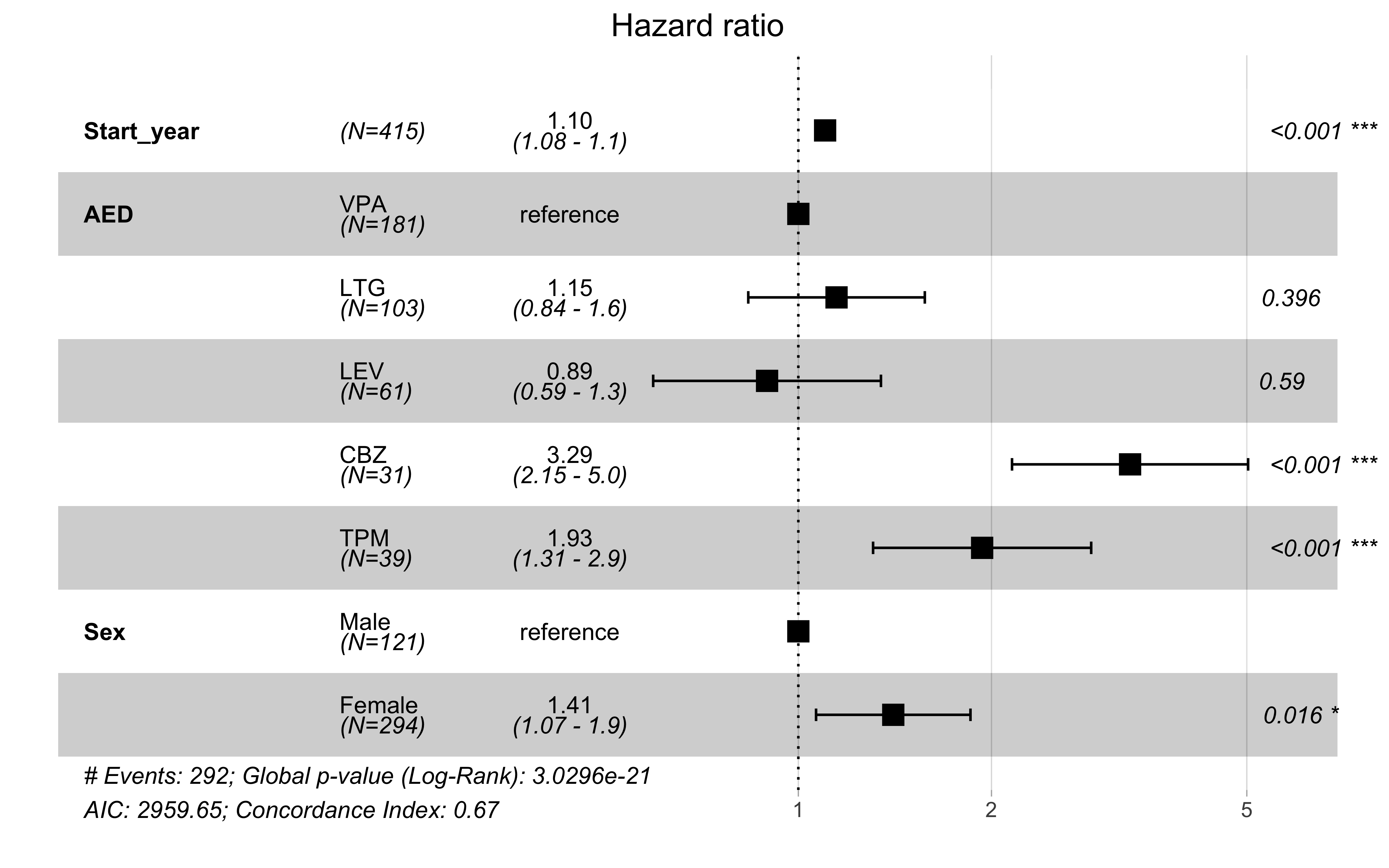


Supplementary Figure 4: Hazard ratios as calculated with the Cox proportional hazards model. The reference level for AEDs is valproate while the reference level for sex is male. The lines mark the 95% confidence interval. P values are displayed on the right.

###

### Reasons for AED discontinuation

The reason for AED discontinuation was unknown in a substantial proportion of trials (31%). Among identified reasons, the most frequent were lack of effectiveness (22.7%), ADR (19.8%), or both lack of effectiveness and ADR (10%). Remission was reported as reason for discontinuation in only 6.7% of trials. Among the AEDs, valproate had the highest rate of discontinuation due to remissions. The reason for discontinuation was recorded as “other” in 10% of trials (Supplementary Table 7).

Supplementary Table 7: Reasons for AED discontinuation

| AED | ADR | Both lack of effectiveness and ADR | Lack of effectiveness | Other | Remission | Unknown |
| --- | --- | --- | --- | --- | --- | --- |
| VPA | 51 (25.1%) | 25 (12.3%) | 16 (7.9%) | 29 (14.3%) | 24 (11.8%) | 58 (28.6%) |
| LTG | 10 (10.2%) | 11 (11.2%) | 40 (40.8%) | 3 (3.1%) | 5 (5.1%) | 29 (29.6%) |
| LEV | 10 (14.9%) | 9 (13.4%) | 17 (25.4%) | 5 (7.5%) | 2 (3.0%) | 24 (35.8%) |
| CBZ | 6 (10.0%) | 0 (0%) | 23 (38.3%) | 8 (13.3%) | 0 (0%) | 23 (38.3%) |
| TPM | 18 (34.0%) | 3 (5.7%) | 13 (24.5%) | 3 (5.7%) | 1 (1.9%) | 15 (28.3%) |

For females, pregnancy-related and “other” reasons for discontinuation were identified for a fifth of trials (Supplementary Table 8). For a total of 19 individual AED trials, a pregnancy or family planning-related reason was stated for discontinuation. Among these, the AED in question was valproate in 15, carbamazepine in two, lamotrigine in one, and levetiracetam in one instance each.

Supplementary Table 8: Reasons for VPA discontinuation among males and females

| Reason | Male | Female |
| --- | --- | --- |
| Adverse reaction | 10 (20.0%) | 41 (26.5%) |
| Both lack of effectiveness and ADR | 10 (20.0%) | 15 (9.7%) |
| Lack of effectiveness | 3 (6.0%) | 13 (8.4%) |
| Other | 0 (0%) | 13 (8.4%) |
| Pregnancy related | 0 (0%) | 19 (12.3%) |
| Remission | 5 (10.0%) | 19 (12.3%) |
| Unknown | 22 (44.0%) | 35 (22.6%) |

### Additional comments for ADRs

The lowest rates of ADRs were observed for carbamazepine, which may have been influenced by missing data for older trials. Also for lamotrigine, the rate of ADRs was also significantly lower than for topiramate or valproate, and discontinuation due to their presence was rare, in keeping with good tolerability. For levetiracetam, the rate of ADRs and discontinuation due to them were higher. Behavioural disorders, including irritability and aggression, emerged as the commonest ADR associated with levetiracetam. The combined rate of behavioural disorders and depression were in keeping with retrospective data of psychiatric adverse effects in a more diverse group of people with epilepsy.^1^

### Valproate dosage

The median maximum daily dose of valproate was significantly lower in trials associated with response compared to failed trials (Mann-Whitney *U* p=0.004; see Supplementary Figure 5).


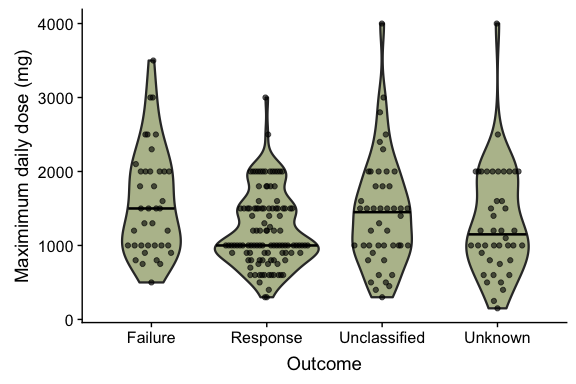


Supplementary Figure 5: Valproate dosage by trial outcome. The median daily dose, marked by a horizontal line, was 1500mg for trials associated with failure (interquartile range (IQR) 1000-2000mg), 1000mg for trials associated with response (IQR 900-1500mg), 1450mg for trials with unclassified outcome (IQR 1000-1800mg), and 1150mg for trials with unknown outcome (825-2000mg).

### Supplementary References:

1. Weintraub D, Buchsbaum R, Resor SR Jr, et al. Psychiatric and behavioral side effects of the newer antiepileptic drugs in adults with epilepsy. Epilepsy Behav 2007;10:105–110.
